# Elevation of Plasma Homocysteine and Minor Hallucinations in Parkinson's Disease: A Cross-Sectional Study

**DOI:** 10.1155/2022/4797861

**Published:** 2022-03-07

**Authors:** Min Zhong, Sha Zhu, Ruxin Gu, Yaxi Wang, Yinyin Jiang, Yu Bai, Xu Jiang, Bo Shen, Jun Yan, Yang Pan, Jun Zhu, Li Zhang

**Affiliations:** ^1^Department of Geriatric Neurology, The Affiliated Brain Hospital of Nanjing Medical University, Nanjing, China; ^2^Department of Biological Sciences, University of Toronto Scarborough, Canada; ^3^Institute of Neuropsychiatric Diseases, The Affiliated Brain Hospital of Nanjing Medical University, Nanjing, China

## Abstract

**Purpose:**

Minor hallucinations (MHs) are the most common psychotic phenomena in Parkinson's disease (PD), and it has important clinical and prognostic implications in PD. Plasma homocysteine (Hcy) has been reported to predict the outcome of PD; whether or not Hcy is associated with MH is not known. We aim to investigate the Hcy level and related factors in patients with PD and MH.

**Methods:**

We conducted a cross-sectional study and included 99 patients with PD, 34 with MH, and 65 without any hallucinations. The clinical and demographic data of the patients with and without hallucinations were compared. Hcy-related clinical factors were also analyzed.

**Results:**

The plasma Hcy level was higher in MH patients than in patients without hallucinations, and the result was more pronounced in male patients than in female patients. Differences were also observed when the groups were divided on the basis of levodopa equivalent daily dose and disease duration. The high Hcy concentration was correlated with some symptoms in patients with MH, including motor dysfunction and nonmotor symptoms, such as symptoms of the gastrointestinal tract, angiocarpy, sleep/fatigue, and poor visuospatial/executive function.

**Conclusions:**

Results indicated a higher plasma Hcy concentration in MH patients than in their counterparts and revealed that Hcy is associated with certain motor and nonmotor symptoms in patients with MH. Hcy may be a marker of MH and have important therapeutic implications in PD.

## 1. Introduction

Parkinson's disease (PD) is the second-largest neurodegenerative disease with clinical manifestations of motor and nonmotor symptoms. As a common nonmotor symptom, minor hallucinations (MHs) have been recognized as a premotor symptom [1, 2] and the most frequently present hallucination in PD [3]. MHs consist of presence hallucinations, passage hallucinations, and visual illusions [4]. With presence hallucination, the patient feels the presence of someone nearby and tends to look around for verification. With passage hallucination, the patient catches a glimpse of a fuzzy shadow passing by, which is often reported as a person or an animal. Visual illusions include kineptosia (seeing still life as moving), pareidolias (seeing human faces or others from complex patterns), and object misidentification illusions (seeing something as another object with a similar shape) [5]. MHs are associated with a high risk of severe psychiatric symptoms, deterioration of cognitive state, or accelerated disease progression [4].

**Figure 1:**
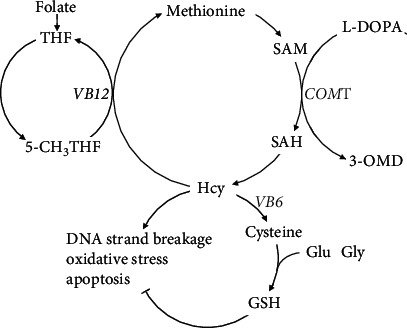
The role of homocysteine in the methionine cycle. In the presence of folate and vitamin B12, homocysteine (Hcy) is converted to methionine. Methionine is converted back to Hcy through a series of reactions under the catalysis of catechol-O-methyltransferase, and this catalytic reaction can reduce levodopa. On the one hand, Hcy can cause synaptic dysfunction and cell death through DNA strand breakage, oxidative stress, and apoptosis. On the other hand, Hcy can be converted to cysteine, which is the raw material of glutathione. Glutathione can reduce the adverse effects of Hcy to a certain extent. 3-OMD: 3-O-methyldopa; 5-CH_3_THF: 5-methyltetrahydrofolate; COMT: catechol-O-methyltransferase; Glu: glutamate; Gly: glycine; GSH: glutathione; Hcy: homocysteine; L-DOPA: levodopa; SAH: s-adenosyl-homocysteine; SAM: s-adenosyl-methionine; THF: tetrahydrofolate; VB12: vitamin B12; VB6: vitamin B6.

Homocysteine (Hcy) is an important intermediate product produced during the metabolism of methionine, known as the methionine cycle. As shown in Figure 1, Hcy and methionine can be converted into each other. This conversion mechanism depends on folate and vitamin B12 (VB12). Under the catalysis of a series of reductases, folate can be activated to 5-methyltetrahydrofolate (5-methylTHF), the main form of folate in serum. The function of 5-methylTHF in this circle is to provide methyl while VB12 is an important source of coenzyme. Levodopa (L-DOPA) and catechol-O-methyltransferase (COMT) are also involved in the methionine cycle [6]. COMT is an enzyme that participates in the metabolism of L-DOPA and other catechol compounds. On the one hand, Hcy can cause oxidative stress and gene expression error, leading to synaptic dysfunction and cell death [7, 8]. On the other hand, Hcy, together with glutamate and glycine, can turn into glutathione, which in turn inhibits the destructive effect of Hcy [9].

High-level Hcy is closely related to vascular diseases; evidence has shown that it also plays an important role in neurodegenerative disorders, such as PD [9]. Patients with PD have relatively high plasma Hcy levels, and the level of Hcy predicts different outcomes [10]. Meanwhile, emerging evidence indicates that Hcy is associated with psychiatric disorders. The risk of schizophrenia increases as the level of Hcy rises [11]. As Hcy is associated with neuropsychiatric diseases, we proposed that Hcy might be related to PD-MH.

Motivated by our previous observation of the plasma Hcy levels of patients with PD presenting MHs, we aim to investigate the Hcy level and related factors in patients with PD and MH.

## 2. Material and Methods

### 2.1. Patients and Study Design

**Figure 2:**
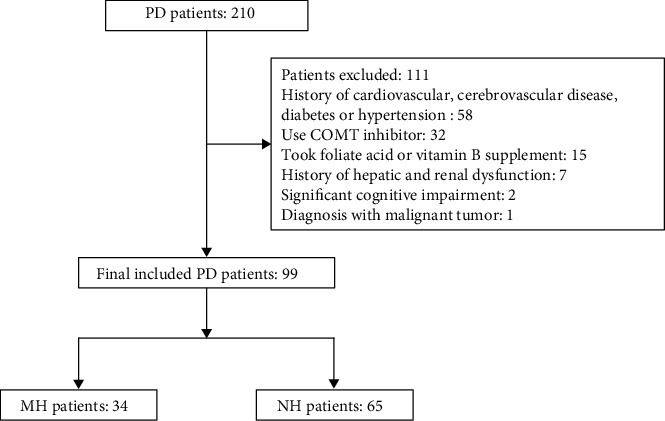
Study flow chart. PD: Parkinson's disease; COMT: catechol-O-methyltransferase; MH: minor hallucination; NH: no hallucinations.

From May 2019 to January 2021, 210 patients with idiopathic PD were consecutively recruited from the Affiliated Brain Hospital of Nanjing Medical University, Nanjing, P. R. China. The diagnosis of PD patients was carried out by at least two movement-disorder specialists on the basis of the PD United Kingdom Brain Bank criteria [12]. The exclusive criteria for this study included the following: (1) significant cognitive impairment, with Montreal Cognitive Assessment (MoCA) score < 20 or a diagnosis of dementia; (2) history of major psychiatric diseases (or use of any antipsychotic medication), malignant tumor, diabetes, hypertension, hepatic and renal dysfunction, or cardiovascular and cerebrovascular disease (all patients had an MRI scan before inclusion in this study); (3) had a chronic or acute infection, took foliate acid or vitamin B supplement, and treated with COMT inhibitor in the last three months before inclusion; and (4) abnormal vision and corrected vision according to Snellen's illiterate “E” chart (worse than 6/12) [1, 13]. Ninety-nine patients were finally included in the study (Figure 2). The Hallucinations and Psychosis item of the Movement Disorder Society-sponsored revision of the Unified Parkinson's Disease Rating Scale (MDS-UPDRS) Part 1 was used as the primary screening tool to assess the presence of MHs. The scores were as follows: 0 = no hallucinations, 1 = minor hallucinations, 2 = formed hallucinations with insight, 3 = formed hallucinations without insight, and 4 = delusions. Any patient with a score of 0 in this item was included in the PD-NH group (*n* = 34). Those with a score of 1 and fulfilled the condition in which MHs occurred steadily and more than once per week in the last three months before the study were enrolled in the PD-MH group (*n* = 65). A questionnaire [14] of 12 items including three aspects (external factors, temporal factors, and content) was used to confirm the presence of MH. The clinical characteristics and plasma levels of Hcy, folate, and VB12 were compared between the MH group and the NH group. According to previous studies [15, 16], we also divided the two groups into subgroups based on gender, levodopa equivalent daily dose (LEDD) [17], and disease duration to assess the Hcy differences.

This study was approved by the local Ethics Committee of the Brain Hospital Affiliated to Nanjing Medical University and was conducted in accordance with the principles outlined in the Declaration of Helsinki. All participants signed informed written consent and provided clinical data.

### 2.2. Clinical Outcomes

The following demographic information was collected using a standard questionnaire: age, gender, marriage, education level, body mass index (BMI), history of smoking and drinking, history of drinking tea and coffee, daily exercise, family history of PD, predominance of motor symptoms, disease duration, and use of antiparkinsonian drugs [14].

Motor disability and disease severity were assessed using the Unified Parkinson's Disease Rating Scale (UPDRS) part III score and the Hoehn and Yahr (H–Y) scale, respectively. H–Y stage ≤ 2 was defined as early stage while H–Y stage > 2 as the advanced stage. The Non-Motor Symptoms Questionnaire (NMS-Quest) was used to measure the nonmotor symptoms in PD [18]. Global cognitive function was evaluated using MoCA [19]. All patients accomplished the PD Sleep Scale (PDSS) to measure sleep quality [20] and the REM sleep behavior disorder Screening Questionnaire (RBDSQ) to further evaluate the occurrence of possible RBD (pRBD). An RBDSQ score of 5 points or more was indicative of pRBD [21]. Emotion evaluation was performed using the Hamilton Anxiety Rating Scale (HAMA) and the Hamilton Rating Scale for Depression (HAMD). Parkinson's Disease Questionnaire-39 (PDQ39) was administered to examine health-related quality of life [22].

### 2.3. Blood Sampling Measurement

After fasting overnight, 3 mL of venous blood was collected from all patients at 7 am. The blood samples were placed on ice and then centrifuged at 3,000 rpm for 10 min to obtain the plasma. The levels of Hcy, folate, and VB12 were measured from plasma. These steps were completed within 2 h. Hcy was determined by enzymatic cycling technology using an Hcy reagent kit (Biosino, 200501, Beijing, China) in an automatic biochemical analyzer (Beckman, AU5800, USA). Enzymatic cycling technology uses enzymes to convert Hcy into a free form, reacts with covalent substrates, and circulates amplification. The final product converts nicotinamide adenine dinucleotide (NADH) into nicotinamide adenine dinucleotide. The concentration of Hcy is detected by measuring the absorbance of NADH. Chemiluminescence microparticle immunoassay technology, which combines magnetic separation technology, chemiluminescence technology, and immunoassay technology, was conducted using the ARCHITECT i system (Abbott, i2000SR, USA) of a folate reagent kit (Abbott, 1P74/08P14, USA) and a B12 reagent kit (Abbott, 7 K61/07P67, USA) to investigate the plasma levels of folate and VB12, respectively. The levels of Hcy, folate, and VB12 were expressed in *μ*mol/L, nmol/L, and pmol/L, respectively.

### 2.4. Statistical Analysis

Our research is mainly a comparison between two groups. In this study, all continuous variables were presented as the mean ± standard deviation (SD). The Kolmogorov–Smirnov test was used to analyze the normal distribution of all variables. When the data conformed to the normal distribution, Student's *t*-test was used; the Mann–Whitney *U* test was conducted otherwise. Categorical variables were shown as percentages and analyzed using a chi-square test and Pearson's and Fisher's exact test. When the data were ordinal, the Mann–Whitney *U* test was used. Correlations between the clinical characteristics and Hcy levels were performed using Spearman's rank correlation coefficient (*r*_*s*_). SPSS 23.0 (IBM Corporation, New York, USA) was used in the data analysis, and GraphPad Prism 8.0 was used to draw histograms. Values of *P* < 0.05 were deemed statistically significant.

## 3. Results

### 3.1. Characteristics of Patients

Table 1 presents the demographic features of the patients. Among the 99 patients, 34 patients [16 males (47.1%) and 18 (52.9%) females] were found to have MHs (PD-MH) while 65 patients [32 males (49.2%) and 33 (50.8%) females] were found not to have any hallucinations (PD-NH). These patients were similar in age, gender, percentage of living alone, education level, BMI, and personal living habits (e.g., history of smoking; drinking alcohol, coffee, or tea; and daily exercise). No significant differences were noted in terms of the family history of PD, predominance of motor symptoms, disease duration, PD medication, LEDD, UPDRS III scores, and modified H–Y stage.

The clinical characteristics of all the patients are summarized in Table 2. The PD-MH patients had significantly higher NMS scores than the PD-NH patients (*P* = 0.001), and the subdomains of the NMS-Quest scale, including gastrointestinal tract, depression/anxiety/anhedonia, sleep/fatigue, and miscellaneous (e.g., diplopia and weight loss), were significantly different between them (*P* = 0.021, 0.019, 0.006, and 0.002, respectively). Among all the nonmotor symptoms of PD, the problems of sleep quality (*P* = 0.028) and pRBD (*P* = 0.04) were more severe in the patients with MHs than in those without. Compared with the patients without any hallucinations, those with MHs had lower scores in PDQ39 (*P* = 0.028) and relatively higher scores in HAMA (*P* = 0.084). No significant differences were noted in the scores of HAMD (*P* = 0.279) and MoCA (*P* = 0.84); however, the scores of MoCA in the visuospatial/executive domain were relatively high in the nonhallucinators (*P* = 0.034). In this study, the plasma level of Hcy was higher in the PD-MH patients than in the PD-NH patients (*P* = 0.001). Meanwhile, the plasma level of folate tended to be higher in the PD-NH group than in the PD-MH group (*P* = 0.053) while no significant differences in VB12 levels were noted between the minor hallucinators and the nonhallucinators.

### 3.2. Comparison of Hcy/Folate/VB12 between Two Groups Based on Gender

**Figure 3:**
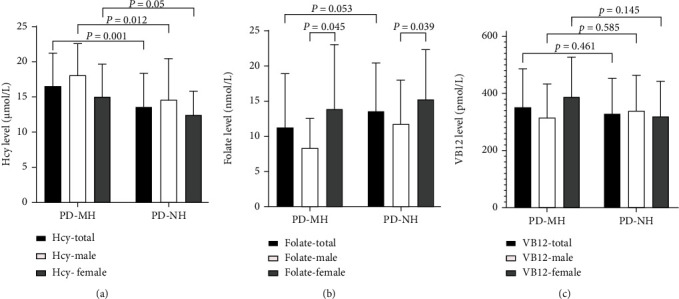
Comparison of Hcy, folate, and vitamin B12 levels between MH and NH patients, according to gender. (a) Comparison of Hcy level between the PD-MH and PD-NH groups. PD-MH (total) vs. PD-NH (total), *P* = 0.001; PD-MH (male) vs. PD-NH (male), *P* = 0.012; PD-MH (female) vs. PD-NH (female), *P* = 0.05. (b) Comparison of folate level between the PD-MH and PD-NH groups. PD-MH (total) vs. PD-NH (total), *P* = 0.053; PD-MH (male) vs. PD-MH (female), *P* = 0.045; PD-NH (male) vs. PD-NH (female), *P* = 0.039. (c) Comparison of vitamin B12 level between the PD-MH and PD-NH groups. PD-MH (total) vs. PD-NH (total), *P* = 0.461; PD-MH (male) vs. PD-NH (male), *P* = 0.585; PD-MH (female) vs. PD-NH (female), *P* = 0.145. Hcy: homocysteine.

When the patients were divided into specific gender groups, the Hcy levels of the males in the two groups significantly differed (*P* = 0.012). Meanwhile, the Hcy levels exhibited an upward trend in the female patients in the PD-MH group relative to those in the PD-NH group (*P* = 0.05). Folate levels displayed significant differences between the male and female patients (*P* = 0.045 for PD-MH, *P* = 0.039 for PD-NH). VB12 levels did not present any differences in the subgroups (Table 3, Figure 3).

### 3.3. Comparison of Hcy Activities according to LEDD and Disease Duration

When the patients were divided into specific groups according to LEDD, our data (Table 4) showed no significant difference between the PD-MH and PD-NH patients, except when the LEDD setting was >0 (*P* = 0.009). When the patients were further divided into three subgroups on the basis of LEDD, we found that plasma levels were significantly higher in the PD-MH patients than in the PD-NH patients in the LEDD > 750 subgroup (*P* = 0.043). The subgroup of 375 ≤ LEDD ≤ 750 was tended to have a high level of Hcy (*P* = 0.078). When disease duration > 5 years, the PD-MH patients had a higher level of Hcy than the PD-NH patients (*P* = 0.002).

### 3.4. Correlations between Hcy Levels and Clinical Assessment Scales in PD-MH Patients

The results of Spearman's correlation analysis among Hcy and the other clinical assessment scales for the PD-MH patients are summarized in Table 5. The plasma Hcy levels were found to be negatively correlated with folate, VB12, and MoCA visuospatial/executive subdomain (*P* = 0.04, 0.014, and 0.036, respectively) and positively correlated with UPDRS III; NMS total score; and NMS gastrointestinal tract, cardiovascular, and sleep/fatigue subdomains (*P* = 0.04, 0.031, 0.016, 0.008, and 0.027, respectively). In the PD-MH patients, no significant correlations were noted between Hcy and age, Hcy and LEDD, Hcy and NMS other subdomains, Hcy and PDSS, Hcy and total MoCA, Hcy and HAMA, Hcy and HAMD, and Hcy and PDQ39.

## 4. Discussion

In this cross-sectional study, we found that apart from the many clinical implications and factors, including gastrointestinal tract, mood, sleep, miscellaneous (e.g., diplopia, weight loss), life quality, and visuospatial and executive ability, Hcy was associated with MHs in the case of consistent baseline data. We found increased plasma Hcy levels in MH patients, especially in men, compared to NH patients. As the disease progresses and the dose of medication increases, the Hcy level of MHs also increases more significantly than that of NH. Significant correlations were observed between plasma Hcy levels and some symptoms in the PD-MH group, including motor dysfunction, cognition subdomain impairment, mood, gastrointestinal tract symptoms, cardiovascular disorder, and sleep quality. To the best of our knowledge, this work is the first to focus on PD-MH patients, explore changes in the plasma levels of Hcy, and evaluate the potential relationships between Hcy levels and certain motor and nonmotor symptoms in patients with MH. Our findings suggested that plasma Hcy may underlie the pathophysiological mechanisms of MHs and could be used to improve health-related life quality of these patients.

MHs are the most frequent and earliest type of psychotic phenomena in PD, and they can appear throughout the course of PD disease, even before the onset of motor symptoms [1, 23]. The early identification of isolated patients with MH as high-risk groups is crucial. MH imposes additional burden on PD patients in terms of nonmotor symptoms and life quality. Lenka et al. proposed that MHs in PD may be just the tip of the iceberg, below which are extensive nonmotor symptoms, and may develop into severe psychiatric symptoms and accelerated disease progression [4]. They are at increased risk of developing dementia or being placed in a nursing facility [24].

Many factors can affect the concentration of Hcy:

- Nutritional factors and lifestyle: excessive consumption of folate and vitamin B or too little intake of these nutrients helps elevate Hcy level. In addition, smoking, alcohol consumption, and physical inactivity are factors associated with elevated plasma Hcy level.
- Age and gender: some studies have suggested a correlation between HHcy and advanced age and male gender [25–27].
- Diseases and medicines: different pathological conditions, such as diabetes, hypertension, malignant tumor, and hepatic and renal insufficiency [28–31], as well as the intake of medicine, such as COMT inhibitor, methotrein, and diuretics, can increase the blood level of Hcy [6].
- Genetic factors: gene defects that affect the expression of various enzymes in the one-carbon cycle affect Hcy [7, 25].

Although many reports have discussed and confirmed the relationships between Hcy and PD or psychiatry disease separately [32], little attention has been given to Hcy and psychiatry symptoms in PD. In the univariate analysis, we found that patients with MHs showed a higher plasma level of Hcy than the nonhallucinators. This result implies that Hcy may participate in the pathological mechanism of MHs. Hcy may affect MHs through different mechanisms [25]. (1) Hcy acts as a glutamate receptor agonist that directly activates glutamate receptors or indirectly activates glutamate receptors through competition with inhibitory neurotransmitters, thereby directing excitatory action on neurons and enhancing calcium influx. Meanwhile, Hcy can induce and even potentiate with some pathological protein aggregation, such as Abeta and tau protein. (2) Hcy induces a decrease in NO and an increase in ROS, and it produces a series of immune cascade reactions. Hcy accelerates the damage of neurons through these mechanisms, thereby causing specific brain areas, such as secondary visual cortex areas, and key areas of the default mode network (right anterior cuneiform lobe, right posterior cingulate cortex, and right parahippocampal gyrus) to reduce the volume of gray matter and functional connectivity changes, which eventually lead to the production of MHs [5, 33]. A previous study denied the relationship between Hcy and hallucinations [16]; such finding is contrary to ours. One possible explanation points to the different conditions for inclusion. In our research, we strictly controlled the influence of the modified factors as much as possible.

Furthermore, we found that the gender difference of Hcy was highly pronounced in the MH patients. Meanwhile, the folate concentration was lower in males than in females. These results are consistent with previous findings [31], but the underlying mechanism is not well known. After menopause, females' Hcy concentrations can increase and converge with those of males [34]. This suggests that gender-specific hormones may be the main reason for gender differences in Hcy concentrations. Although overall the duration of illness and the dosage of parkinsonism medicine were not significantly different between the two groups, the Hcy level between the two groups was significantly different when the two factors were at a high level. This result may indicate that as PD progresses, the Hcy level of MH patients will be higher than that of patients without hallucinations and will further aggravate the condition.

In the MH patients, Hcy showed positive correlations with UPDRS III and NMS (gastrointestinal tract and cardiovascular symptoms, sleep/fatigue) and negative correlations with visuospatial/executive function. This result strongly implied that Hcy influences motor and nonmotor symptoms in MH patients. Our results are consistent with the findings associating Hcy with motor function and cognitive decline [35]. Increased plasma Hcy is an established risk factor for cardiovascular disease and cognition [36–38]. In contrast to previous trials reporting that Hcy is inversely correlated with semantic verbal fluency and verbal memory function [39], we found that Hcy affected selected cognitive aspects of the visuospatial/executive function in MH patients. We suspected that elevated Hcy might cause the dysfunction of the posterior association neocortex through oxidative stress, which in turn leads to visuospatial disorder. At present, no evidence proves how elevated Hcy causes dysfunction in the posterior association neocortex, while other brain regions are not disturbed; this novel finding should be considered tentative and requires replication. The sleep problems of patients with PD, including sleep structure disorder, insomnia, excessive daytime sleepiness, and RBD, are prominent [40], especially in patients with MHs. These sleep disorders involve many brain regions and nuclei. Hcy is positively correlated with sleep disorders. Thus, hyperhomocysteinemia and sleep disorders are more than just comorbidities in patients with PD with MHs. Existing studies have shown that Hcy can reduce the secretion of melatonin [41]. This result may partly explain sleep disorders in MH patients. The specific mechanism behind this condition remains unknown, and further research is needed.

Consistent with previous research, our study demonstrated that Hcy is reversely correlated with folate and VB12 [7, 38]. Supplementing folic acid and vitamin B can reduce plasma Hcy concentration, thus providing novel ideas for improving MH symptoms, which may alter disease trajectory. Meanwhile, Hcy is positively correlated with gastrointestinal tract symptoms. The high concentrations of Hcy may be partly due to reduced gastrointestinal absorption in patients with MHs. VB12 supplementation can reduce the development of neuropathy/myelopathy and may indirectly support our result [42].

Studies about the relationship between Hcy and LEDD have yielded inconsistent results. Some researchers believe that they are correlated [39], whereas others deny such finding [43]. In our study, we did not find a relationship between plasma Hcy levels and LEDD or the duration of disease.

Some limitations existed in our research. First, the number of patients was relatively small because of the strict inclusion criteria, and a referral bias might have influenced the results. Second, this work is a cross-sectional study. In a certain period of time, the continuous observation of patients' symptoms and plasma Hcy concentration is beneficial to understand the relationship and changes between the two. Third, the severity of certain nonmotor symptoms was not assessed using specific and detailed scales.

## 5. Conclusion

MHs exert important clinical and prognostic effects on PD. Despite similar demographics, medications, and motor symptoms, PD patients with MHs showed a greater burden of nonmotor symptoms and a declined quality of life compared with PD patients without any hallucinations. Plasma Hcy levels were relatively high in MH patients. The high levels of Hcy may be a cause of other comorbid symptoms in these patients, and it explained the pathophysiological mechanisms of disease development. Hcy may be a marker of MHs and will increase the detection rate of MHs, thereby making up for the shortcomings of the scale. Whether Hcy can be a possible target in the treatment of MHs requires further study.

## Figures and Tables

**Table 1:** Demographic parameters in the PD-MH and PD-NH patients.

		PD-MH	PD-NH	*χ* ^2^/*t*/*Z* value	*P*
Number (%)		34 (34.3%)	65 (65.7%)		
Age (y), mean ± SD		67.38 ± 7.32	69.55 ± 10.15	934.5	0.208^a^
Gender (%)	Male	16 (47.1%)	32 (49.2%)	0.042	0.837^b^
	Female	18 (52.9%)	33 (50.8%)		
Percentage of live alone (%)		2 (5.9%)	8 (12.3%)	1.005	0.487^b^
Education (%)	Illiterate	1 (2.9%)	13 (20.0%)	944.5	0.19^a^
	Primary school	3 (8.8%)	6 (9.2%)		
	Middle school	23 (67.6%)	32 (49.2%)		
	College or above	7 (20.6%)	14 (21.5%)		
BMI, mean ± SD		23.75 ± 3.99	23.22 ± 3.28	0.701	0.485^c^
Smoker (%)		8 (23.5%)	16 (24.6%)	0.014	0.905^b^
Alcohol intake (%)		3 (8.8%)	7 (10.8%)	0.092	1^b^
Drinking tea (%)		7 (20.6%)	16 (24.6%)	0.203	0.652^b^
Drinking coffee (%)		4 (11.8%)	4 (6.2%)	0.937	0.441^b^
Daily exercise (%)		19 (55.9%)	41 (63.1%)	0.484	0.487^b^
Family history of PD (%)		1 (2.9%)	9 (13.8%)	2.894	0.157^b^
Predominance of motor symptoms (%)		4 (11.8%)	8 (12.3%)	1.531	0.216^b^
	Left	11 (32.4%)	33 (50.8%)		
	Right	19 (55.9%)	24 (36.9%)		
Disease duration (y), mean ± SD		6.47 ± 4.84	5.05 ± 4.11	904.5	0.137^a^
					
PD treatment (%)	Benzhexol	0 (0%)	1 (1.5%)	0.523	1^b^
	Amantadine	8 (23.5%)	7 (10.8%)	2.827	0.093^b^
	MAO-B inhibitor	5 (14.7%)	8 (12.3%)	0.111	0.76^b^
	Dopamine agonist	22 (64.7%)	30 (46.2%)	3.081	0.079^b^
LEDD (mg), mean ± SD		370.44 ± 279.59	303.96 ± 308.52	932	0.198^a^
UPDRS III, mean ± SD		30.24 ± 15.68	28.95 ± 12.09	1092.5	0.927^a^
Modified H–Y (%)	1—2	8 (23.5%)	24 (36.9%)	1.831	0.176^b^
	2.5—5	26 (76.5%)	41 (63.1%)		

^a^Mann-Whitney *U* test. ^b^Chi-square test. ^c^Student's *t*-test. SD: standard deviation; PD: Parkinson's disease; MH: minor hallucination; NH: no hallucinations; BMI: body mass index; LEDD: Levodopa equivalent daily dose; UPDRS III: the Unified Parkinson's Disease Rating Scale part III; H–Y stage: Hoehn and Yahr stage.

**Table 2:** Clinical characteristics of the PD-MH and PD-NH patients.

Variable		PD-MH	PD-NH	*χ* ^2^/*Z* value	*P*
NMS-Quest, mean ± SD		13.91 ± 5.33	10.31 ± 4.59	661.5	**0.001** ^a^
	Gastrointestinal tract, mean ± SD	3.09 ± 1.76	2.28 ± 1.41	798.5	**0.021** ^a^
	Urinary tract, mean ± SD	1.06 ± 0.85	0.95 ± 0.86	1030.5	0.56^a^
	Sexual function, mean ± SD	0.62 ± 0.89	0.65 ± 0.89	1087	0.876^a^
	Cardiovascular, mean ± SD	0.88 ± 0.59	0.72 ± 0.67	949	0.2^a^
	Apathy/attention/memory, mean ± SD	1.65 ± 1.07	1.34 ± 1.07	933	0.19^a^
	Depression/anxiety/anhedonia, mean ± SD	1.18 ± 0.87	0.74 ± 0.85	808	**0.019** ^a^
	Sleep/fatigue, mean ± SD	2.85 ± 1.35	2.14 ± 1.21	742.5	**0.006** ^a^
	Pain (unrelated to other causes), mean ± SD	0.41 ± 0.50	0.42 ± 0.50	1101	0.972^a^
	Miscellaneous (e.g., diplopia), mean ± SD	1.65 ± 0.98	1.00 ± 0.98	701.5	**0.002** ^a^
PDSS, mean ± SD		98.19 ± 34.05	112.71 ± 27.85	806	**0.028** ^a^
Modified RBDSQ (%)	<5	22(64.7%)	54(83.1%)	4.224	**0.04** ^b^
	≥5	12(35.3%)	11(16.9%)		
MoCA, mean ± SD		27.59 ± 2.04	27.60 ± 2.21	1078	0.84^a^
	Visuospatial/executive, mean ± SD	4.06 ± 0.92	4.46 ± 0.66	842	**0.034** ^a^
	Naming, mean ± SD	3.00 ± 0.00	2.98 ± 0.12	1088	0.47^a^
	Attention, mean ± SD	5.82 ± 0.39	5.58 ± 0.86	1018	0.369^a^
	Language, mean ± SD	2.85 ± 0.36	2.95 ± 0.21	993.5	0.082^a^
	Abstraction, mean ± SD	1.91 ± 0.29	1.82 ± 0.46	1029.5	0.342^a^
	Delayed memory, mean ± SD	3.97 ± 1.03	3.85 ± 1.08	1037.5	0.602^a^
	Orientation, mean ± SD	5.97 ± 0.17	5.95 ± 0.28	1103	0.96^a^
HAMA, mean ± SD		7.85 ± 5.15	6.05 ± 4.47	871	0.084^a^
HAMD, mean ± SD		8.44 ± 5.03	7.25 ± 4.60	958.5	0.279^a^
PDQ39, mean ± SD		52.38 ± 26.29	40.11 ± 24.61	806	**0.028** ^a^
Hcy, mean ± SD		16.56 ± 4.78	13.57 ± 4.87	671.5	**0.001** ^a^
Folate, mean ± SD		11.36 ± 7.71	13.61 ± 6.89	842	0.053^a^
VB12, mean ± SD		350.73 ± 134.45	328.59 ± 122.73	1005	0.461^a^

^a^Mann-Whitney *U* test. ^b^Chi-square test. ^c^The scores were calculated from the NMS-Quest total score excluding the hallucinations/delusions domain score. ^d^Significant results are highlighted in bold (*P* < 0.05). SD: standard deviation; PD: Parkinson's disease; MH: minor hallucination; NH: no hallucinations; NMS-Quest: Non-Motor Symptoms Questionnaire; PDSS: The PD Sleep Scale; RBDSQ: the REM sleep behavior disorder Sreening Questionnaire; MOCA: Montreal Cognitive Assessment; HAMA: Hamilton Anxiety Rating Scale; HAMD: Hamilton Depression Rating Scale. PDQ39: Parkinson's Disease Questionnaire-39; Hcy: homocysteine; VB12: vitamin B12.

**Table 3:** Comparison of Hcy, folate, and vitamin B12 levels between MH and NH patients based on gender.

Variable		PD-MH	PD-NH	PD-MH vs. PD-NH		PD-MH (male) vs. PD-MH (female)		PD-NH (male) vs. PD-NH (female)	
				*Z* value	*P*	*Z* value	*P*	*Z* value	*P*
Hcy (*μ*mol/L)	Male	18.23 ± 4.44	14.67 ± 5.88	140.5	**0.012** ^a^	89	0.058^a^	430	0.198^a^
	Female	15.07 ± 4.69	12.50 ± 3.39	197.5	0.05^a^				
Folate (nmol/L)	Male	8.39 ± 4.19	11.86 ± 6.19	171	0.063^a^	86	**0.045** ^a^	370.5	**0.039** ^a^
	Female	14.00 ± 9.18	15.31 ± 7.20	257	0.43^a^				
VB12 (pmol/L)	Male	313.45 ± 118.45	337.95 ± 125.35	231	0.585^a^	105	0.178^a^	466.5	0.42^a^
	Female	383.86 ± 142.27	319.52 ± 121.36	223	0.145^a^				

^a^Mann-Whitney *U* test. ^b^Significant results are highlighted in bold (*P* < 0.05). ^c^Data was shown as the mean ± SD. SD: standard deviation; PD: Parkinson's disease; MH: minor hallucination; NH: no hallucinations; Hcy: homocysteine; VB12: vitamin B12.

**Table 4:** Comparison of Hcy according to LEDD and disease duration.

Variable		PD-MH	PD-NH	PD-MH vs. PD-NH	
				*Z* value	*P*
Hcy (*μ*mol/L)	LEDD = 0	16.25 ± 6.77	12.90 ± 3.23	33	0.186^a^
	LEDD > 0	16.61 ± 4.51	13.91 ± 5.53	395	**0.009** ^a^
	0 < LEDD < 375	15.87 ± 4.82	13.83 ± 5.83	97	0.235^a^
	375 ≤ LEDD ≤ 750	16.77 ± 4.79	14.37 ± 5.80	70.5	0.078^a^
	LEDD > 750	18.52 ± 2.42	12.15 ± 2.11	1	**0.043** ^a^
	Disease duration ≤ 5y	15.47 ± 5.19	13.97 ± 5.27	253.5	0.22^a^
	Disease duration > 5y	17.41 ± 4.38	12.78 ± 3.97	91	**0.002** ^a^

^a^ Mann-Whitney *U* test. ^b^Significant results are highlighted in bold (*P* < 0.05). ^c^Data was shown as the mean ± SD. SD: standard deviation; PD: Parkinson's disease; MH: minor hallucination; NH: no hallucinations; Hcy: homocysteine; LEDD: Levodopa equivalent daily dose.

**Table 5:** Correlations between Hcy and clinical parameters.

Variable	*r*_*s*_	*P*
Age	0.125	0.480
LEDD	0.148	0.405
UPDRS III	0.354	**0.040**
NMS-Quest	0.371	**0.031**
Gastrointestinal tract	0.408	**0.016**
Urinary tract	0.236	0.179
Sexual function	-0.107	0.545
Cardiovascular	0.444	**0.008**
Apathy/attention/memory	0.306	0.078
Depression/anxiety/anhedonia	-0.078	0.660
Sleep/fatigue	0.38	**0.027**
Pain (unrelated to other causes)	-0.225	0.200
Miscellaneous (e.g., diplopia and weight loss)	0.021	0.907
PDSS	-0.212	0.229
MoCA	-0.103	0.563
Visuospatial/executive	-0.361	**0.036**
Naming	/	/
Attention	0.165	0.351
Language	-0.284	0.104
Abstraction	0.259	0.139
Delayed memory	0.08	0.651
Orientation	-0.009	0.960
HAMA	-0.02	0.912
HAMD	0.074	0.679
PDQ39	0.287	0.100
Folate	-0.476	**0.004**
VB12	-0.417	**0.014**

Significant results are highlighted in bold (*P* < 0.05). The scores were calculated from the NMS-Quest total score excluding the hallucination/delusion domain score. *r*_*s*_: Spearman's rank correlation coefficient; LEDD: Levodopa equivalent daily dose; UPDRS III: the Unified Parkinson's Disease Rating Scale part III; NMS-Quest: Non-Motor Symptoms Questionnaire; PDSS: The PD Sleep Scale; MOCA: Montreal Cognitive Assessment; HAMA: Hamilton Anxiety Rating Scale; HAMD: Hamilton Depression Rating Scale. PDQ39: Parkinson's Disease Questionnaire-39; VB12: vitamin B12.

## Data Availability

The data supporting the findings of this study are included in the article; further inquiries can be directed to the corresponding authors.
